# Preparation of Au-polydopamine functionalized carbon encapsulated Fe_3_O_4_ magnetic nanocomposites and their application for ultrasensitive detection of carcino-embryonic antigen

**DOI:** 10.1038/srep21017

**Published:** 2016-02-12

**Authors:** Lei Ji, Tao Yan, Yan Li, Jian Gao, Qi Wang, Lihua Hu, Dan Wu, Qin Wei, Bin Du

**Affiliations:** 1 Key Laboratory of Chemical Sensing & Analysis in Universities of Shandong, School of Chemistry and Chemical Engineering, University of Jinan, Jinan 250022, China; 2 School of Material Science and Engineering, University of Jinan, Jinan 250022, P.R. China

## Abstract

A novel carbon encapsulated Fe_3_O_4_ nanoparticles embedded in two-dimensional (2D) porous graphitic carbon nanocomposites (Fe_3_O_4_@C@PGC nanocomposites) were synthesized by situ synthesis strategy, which provided a sensor platform owing to a large aspect ratio and porous structure. Polydopamine (PDA) were modified on the surface of Fe_3_O_4_@C@PGC nanocomposites through self-polymerization of dopamine, acting as both the reductant and template for one-step synthesis of gold nanoparticles. The prepared Au/PDA/Fe_3_O_4_@C@PGC nanocomposites show ferromagnetic features, extremely excellent electron transfer, large specific surface area and excellent dispersing property. These are conducive to the electrochemical signal output and the immobilization of antibody. In this work, a highly label-free sensitive magnetic immunosensor was developed based on Au/PDA/Fe_3_O_4_@C@PGC nanocomposites for the detection of carcino-embryonic antigen (CEA). The magnetic glassy carbon electrode was used to fix the Au/PDA/Fe_3_O_4_@C@PGC nanocomposites with the help of magnetic force. Under the optimal conditions, the immunosensor exhibited a wide linear range (0.001 ng/mL–20.0 ng/mL), a low detection limit (0.33 pg/mL), good reproducibility, selectivity and acceptable stability. The proposed sensing strategy may provide a potential application in the detection of other cancer biomarkers.

Carcino-embryonic antigen (CEA) is a highly glycosylated protein found primarily in the apical membrane of enterocytes and a useful tumor marker of colorectal adenocarcinoma^1^. Evaluation of serum CEA levels has a very important significance during the diagnosis and tracking of patients having undergone curative resection^2^. Therefore, developing new convenient, rapid and accurate analytical method is of crucial importance for the detection of CEA. In recent years, many methods were reported for the detection of CEA, including capillary electrophoresis-chemiluminescence immunoassay^3^, colorimetric immunoassay^4^, fluorescent immunoassay^5^, electrochemiluminescence immunoassay^6^ and electrochemical immunoassay^2^. Compared with the above methods, electrochemical immunoassay was widely researched due to its easy operation, high sensitivity, low cost and miniaturization. In this work, we fabricated a novel label-free electrochemical immunosensor based on the magnetic nanoparticles as platform for the sensitive detection of CEA.

Carbon-encapsulated Fe_3_O_4_ magnetic nanoparticles recently have attracted much scientific interest in environmental area and electrochemical fields owing to high magnetic separation efficiency and specific functional modifications^7^,^8^. Carbon encapsulated Fe_3_O_4_ nanoparticles embedded in two-dimensional (2D) porous graphitic carbon nanocomposites (Fe_3_O_4_@C@PGC nanocomposites) is prepared as a high-rate magnetic conductive material in this work. It shows super-high rate capability and extremely excellent electron transfer, exhibiting great potential as substrate materials for electrochemical immunosensor. The presence of thin onion-like carbon shells avoids the direct exposure of Fe_3_O_4_ to the substrate solution and keeps the stability of the performance of Fe_3_O_4_ structure^9^.

Dopamine (DA) is a naturally occurring compound with catechol and amine moieties, which owns extremely strong adhesive properties. Therefore, DA is selected as binding agents for forming thin, surface-adherent films onto the surface of inorganic and organic materials by secondary reactions^10^,^11^,^12^,^13^. In this work, polydopamine/Fe_3_O_4_@C@PGC nanocomposites (PDA/Fe_3_O_4_@C@PGC nanocomposites) were obtained through self-polymerization of DA in the solution, and the thickness of the polydopamine shell was controllable. Then, gold nanoparticles were one-step synthesized by as-prepared PDA/Fe_3_O_4_@C@PGC nanocomposites which acted as both the reductant and template. The obtained Au/PDA/Fe_3_O_4_@C@PGC nanocomposites show good electrical conductivity, large specific surface area and good dispersion.

In this paper, a novel label-free magnetic electrochemical immunosensor was developed based on Au/PDA/Fe_3_O_4_@C@PGC nanocomposites modified magnetic glassy carbon electrode (MGCE). Using CEA as model analyte, the sensitive detection of CEA was demonstrated based on the peak current change of potassium ferricyanide (K_3_[Fe(CN)_6_]) before and after the antigen-antibody reaction. The detection sensitivity of the fabricated immunosensor was further increased due to the large specific surface area and good electrical conductivity of the Au/PDA/Fe_3_O_4_@C@PGC nanocomposites. The simple and easy immunoassay has great prospect in the ultrasensitive detection of other cancer biomarkers.

## Experimental

### Apparatus and reagents

CEA antibody (Ab) and CEA are obtained from Beijing Dingguo Changsheng Biotechnology Co. Ltd. (China). Bovine serum albumin (BSA) is purchased from Sigma-Aldrich (Beijing, China). All other chemical reagents are analytical reagents grade and directly used without further purification.

All electrochemical measurements are achieved on a CHI 760D electrochemical workstation (Shanghai Chenhua Instrument Co. Ltd., China). Transmission electron microscope (TEM) images are recorded by a JEM-100C X II microscope (JEOL, Japan). Scanning electron microscope (SEM) is obtained from JSM-6700F micro-scope (JEOL, Japan). X-Ray Powder Diffraction (XRD) is performed with D8 advance X-ray diffractometer (Bruker AXS, Germany).

### Synthesis of 2D Fe_3_O_4_@C@PGC nanocomposites

2D Fe_3_O_4_@C@PGC nanocomposites are prepared according to a reported method^9^. Fe(NO_3_)_3_·9H_2_O (0.73 g), glucose (2 g) and NaCl (15 g) are added into 10 mL of ultrapure water. Then the mixed solution is dried at 80 °C for 24 h. The prepared solids are heated at 750 °C for 2 h in the argon atmosphere. After lower temperature to 250 °C, the solids are continuously calcined for 6 h under air. Once cooled down to room temperature, the obtained solids are washed with ultrapure water until no chloride ion is detected. Finally, the resulting solids are dried at 50 °C.

### Synthesis of PDA/Fe_3_O_4_@C@PGC nanocomposites

50 mg of Fe_3_O_4_@C@PGC is dispersed in 100 mL of 50 mM Tris-HCl solution (pH = 8.5) by sonication for 30 min. Afterwards, 50 mg of dopamine hydrochloride is added and mechanically stirred for 3 h. Subsequently the PDA/Fe_3_O_4_@C@PGC nanocomposites are separated from the suspension by extremely effective magnet. The obtained solids are dried at 50 °C.

### Preparation of the Au/PDA/Fe_3_O_4_@C@PGC nanocomposites

10 mL of PDA/Fe_3_O_4_@C@PGC (1.4 mg/mL) solution and 300 μL of HAuCl_4_ (10 mg/mL) are mixed and the mixture is diluted to 250 mL with ultrapure water. The mixture is mechanically stirred at room temperature for 4 h. After reaction, Au/PDA/Fe_3_O_4_@C@PGC nanocomposites are separated from the suspension by extremely effective magnet and washed with ultrapure water for 3 times. The obtained solids are dried at 50 °C.

### Fabrication of the immunosensor

**Figure 1:**
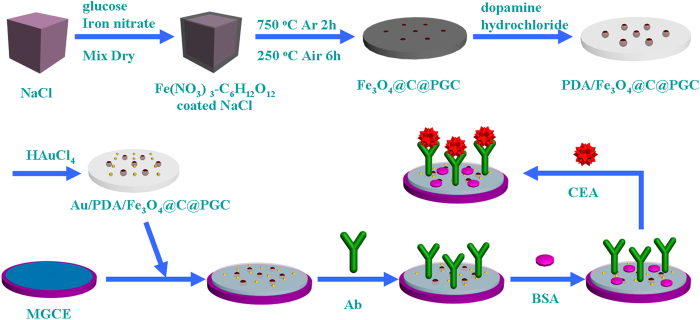
The fabrication of the immunosensor.

Figure 1 displayed the fabrication procedure of the immunosensors. A MGCE with 4-mm diameter was polished with alumina powder (1.0, 0.3 and 0.05 μm) and then thoroughly cleaned before use. To capture Ab, 6 μL of Au/PDA/Fe_3_O_4_@C@PGC solution (1.0 mg/mL) was fixed on the surface of MGCE by the magnetism. Then 6 μL of Ab was anchored on the Au/PDA/Fe_3_O_4_@C@PGC by the physical absorption and the chemical bonding of Au-N^14^ or Au-S^15^,^16^,^17^ bonds. After 1 h of incubation, 3 μL of BSA (1 wt%) was modified onto the MGCE for blocking the nonspecific binding sites. Finally, 6 μL of CEA was added onto the electrode surface and incubated for 1 h. After washing carefully with PBS, the electrode was ready for measurement.

## Results and Discussion

### Characterization of the Au/PDA/Fe_3_O_4_@C@PGC nanocomposites

**Figure 2:**
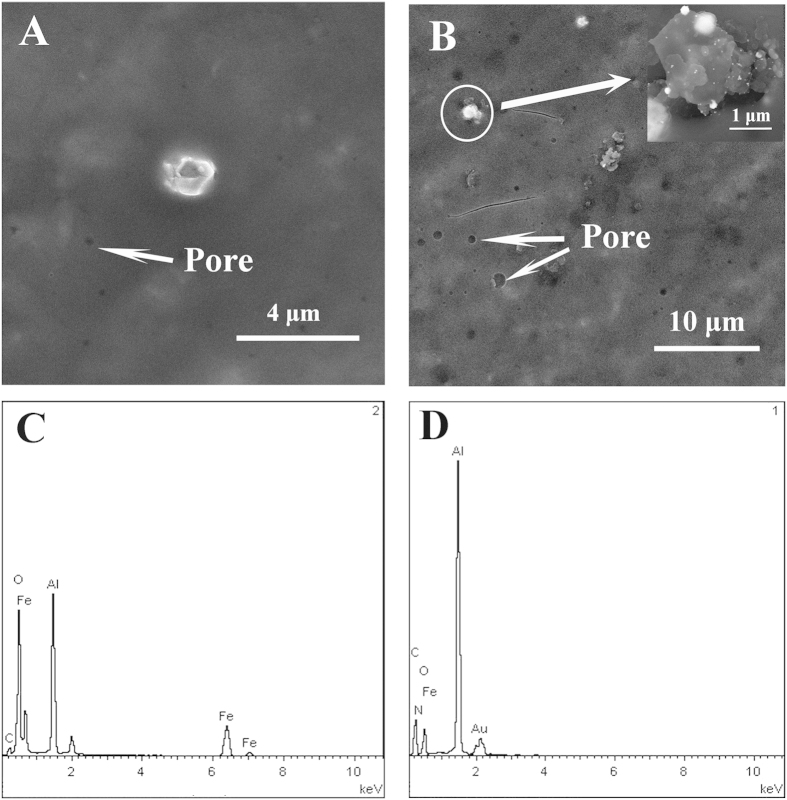
The SEM image of the PDA/Fe_3_O_4_@C@PGC nanocomposites (**A**) and the Au/PDA/Fe_3_O_4_@C@PGC nanocomposites (**B**); The EDX spectrum of PDA/Fe_3_O_4_@C@PGC nanocomposites (**C**) and Au/PDA/Fe_3_O_4_@C@PGC nanocomposites (**D**).

The typical low-magnification SEM images and EDX spectrums of PDA/Fe_3_O_4_@C@PGC nanocomposites and Au/PDA/Fe_3_O_4_@C@PGC nanocomposites were showed in the Fig. 2. SEM image of the synthesized PDA/Fe_3_O_4_@C@PGC nanocomposites presented multi-pore structure with relative smooth surface (Fig. 2(A)). In addition, the PDA/Fe_3_O_4_@C@PGC nanocomposites with a large aspect ratio were well-dispersed in the sample^9^. After modified by Au nanoparticles, Au/PDA/Fe_3_O_4_@C@PGC nanocomposites kept original structure of PDA/Fe_3_O_4_@C@PGC nanocomposites (Fig. 2(B)). The inset of the Fig. 2(B) showed the Au nanoparticles were immobilized on the surface of the PDA/Fe_3_O_4_@C@PGC nanocomposites. The EDX spectrum of PDA/Fe_3_O_4_@C@PGC nanocomposites (Fig. 2(C)) and Au/PDA/Fe_3_O_4_@C@PGC nanocomposites (Fig. 2(D)) were compared and the result was in good agreement with the SEM image.

**Figure 3:**
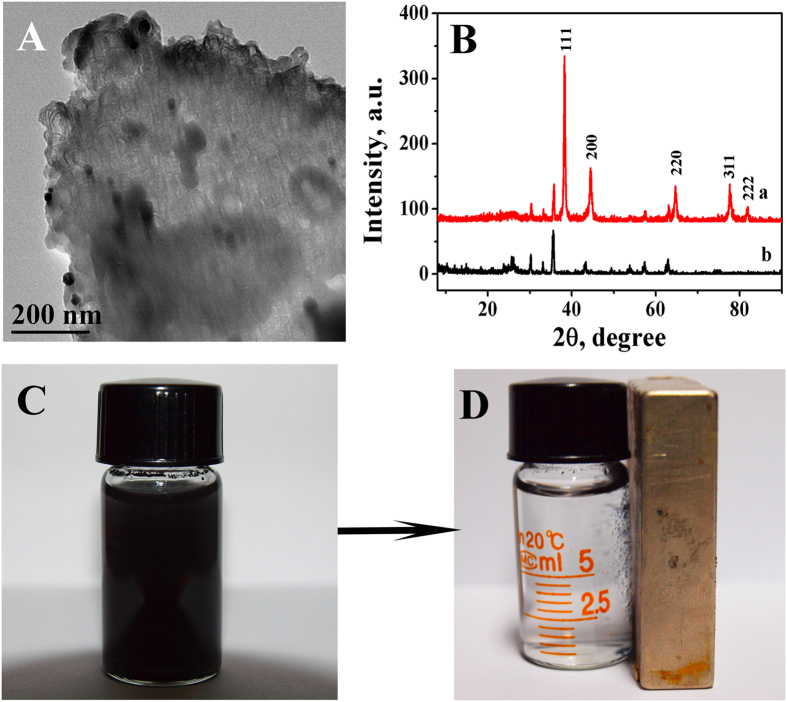
(**A**) The TEM image of the Au/PDA/Fe_3_O_4_@C@PGC nanocomposites; (**B**) the typical XRD patterns of the Au/PDA/Fe_3_O_4_@C@PGC nanocomposites (curve a) and PDA/Fe_3_O_4_@C@PGC nanocomposites (curve b); (**C**) the dispersibility and (**D**) magnetism of Au/PDA/Fe_3_O_4_@C@PGC nanocomposites in ultrapure water.

The Au/PDA/Fe_3_O_4_@C@PGC nanocomposites were further characterized by the TEM (Fig. 3(A)). The result showed these Fe_3_O_4_ nanoparticles were entirely encapsulated by thin onion-like carbon layers and Au nanoparticles were distributed on the surface of PDA/Fe_3_O_4_@C@PGC nanocomposites. In addition, Fig. 3(B) showed typical XRD patterns of the Au/PDA/Fe_3_O_4_@C@PGC nanocomposites (curve a) and PDA/Fe_3_O_4_@C@PGC nanocomposites (curve b). In comparison to the XRD diffraction of the PDA/Fe_3_O_4_@C@PGC nanocomposites, five additional peaks at 38°, 43°, 65°, 78° and 82° which represented the Bragg reflections from (111), (200), (220), (311) and (222) planes of Au were observed (JCPDS card No. 65–2870), showing clearly the existence of Au nanoparticles in the Au/PDA/Fe_3_O_4_@C@PGC nanocomposites.

Figure 3(C) showed the dispersibility of Au/PDA/Fe_3_O_4_@C@PGC nanocomposites in ultrapure water. Owing to the introduction of hydrophilic group of DA, Au/PDA/Fe_3_O_4_@C@PGC nanocomposites can be dispersed to form black dispersion and remain suspended in aqueous solution for 30 min. The Au/PDA/Fe_3_O_4_@C@PGC nanocomposites could be drawn to the sidewall from the solution by applying a magnet beside the vial (Fig. 3(D)), which illustrated that Au/PDA/Fe_3_O_4_@C@PGC nanocomposites have great magnetism.

### Characterization of the immunosensor

**Figure 4:**
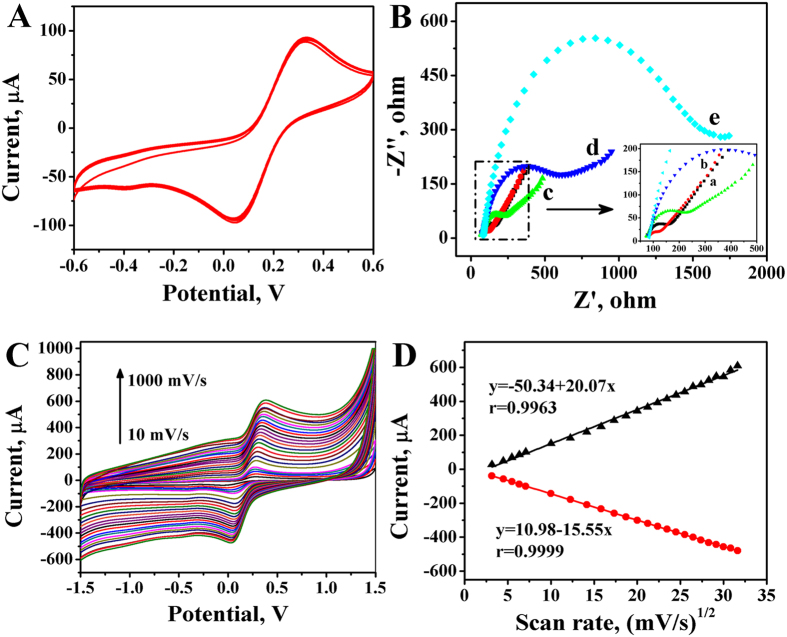
(**A**) 20 successive scans of the Au/PDA/Fe_3_O_4_@C@PGC nanocomposites modified electrode in 5 mmol/L K_3_[Fe(CN)_6_]. Scan rate: 100 mV/s; (**B**) EIS obtained for different modified electrodes in [Fe(CN)_6_]^3-^/[Fe(CN)_6_]^4-^ containing 0.1 M KCl solution (a) GCE, (b) Au/PDA/Fe_3_O_4_@C@PGC/GCE, (c) Ab/Au/PDA/Fe_3_O_4_@C@PGC/GCE, (d) BSA/Ab/Au/PDA/Fe_3_O_4_@C@PGC/GCE, (e) CEA/BSA/Ab/Au/PDA/Fe_3_O_4_@C@PGC/GCE; (**C**) CVs of the modified electrode at different scan rates: 10, 20, 30, 40, 50, 100, 150, 200, 250, 300, 350, 400, 450, 500, 550, 600, 650, 700, 750, 800, 850, 900, 950 and 1000 mV/s in K_4_[Fe(CN)_6_] (1 mM) containing KCl (0.1 M); (**D**) Plots of currents peak as a function of square root of scan rate.

The performance of the immunosensor was also studied under continuous scans as shown in Fig. 4(A). The electrode was scanned successfully for 20 cycles in K_3_[Fe(CN)_6_] solution (5 mmol/L) at 100 mV/s and no observable change of peak current and position was found, illustrating that the reaction between the electrode surface and the substances are not detached and diffusing into the solution during the experiment.

Electrochemical impedance spectroscopy (EIS) was used to research the impedance changes of the electrode surface during the electrode modification process. As shown in Fig. 4(B), bare MGCE showed a very small semicircle diameter (curve a), suggesting a diffusion limiting step of the electrochemical process. Owing to high conductivity and electronic transmission capacity of Au/PDA/Fe_3_O_4_@C@PGC nanocomposites, the semicircle diameter decreased slightly after modified Au/PDA/Fe_3_O_4_@C@PGC nanocomposites (curve b). The charge-transferring resistance (Ret) values increased with addition of Ab_1_ (curve c), indicating that the protein film blocked the electron transfer between substrate solution and MGCE. Similarly, after the immobilization of BSA (curve d) and CEA (curve e), Ret further increased because the additions resist the electron-transfer kinetics of the probe at the electrode interface. These results also demonstrated the biosensor has been fabricated successfully.

The effect of different scan rate on the performance of the fabricated immnosensor was investigated in K_4_[Fe(CN)_6_] (1 mM) containing KCl (0.1 M). Figure 4(C) showed the current response of the cyclic voltammetry with the scan rate range from 10 mV/s to 1000 mV/s. It is found that the magnitude of both anodic and cathodic current response linearly dependent on the square root of scan rate in Fig. 4(D). The observed linear relation and well-defined stable redox peaks suggest that it was a diffusion-controlled electrochemical and quasi-reversible process^18^.

### Optimization of detection conditions

**Figure 5:**
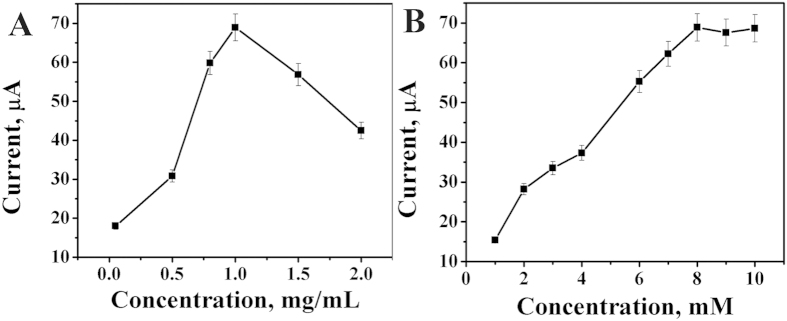
The optimization of experimental conditions with Au/PDA/Fe_3_O_4_@C@PGC concentration (**A**) and K_4_[Fe(CN)_6_] concentration (**B**).

In order to obtain the best analytical performance of the fabricated immunosensor, the concentration of Au/PDA/Fe_3_O_4_@C@PGC nanocomposites has been optimized in Fig. 5(A). Owing to the improvement of electron transfer ability, the current response increased with the increasing concentrations of Au/PDA/Fe_3_O_4_@C@PGC nanocomposites. However, when the concentration of Au/PDA/Fe_3_O_4_@C@PGC nanocomposites was higher than the optimal concentration, the current response decreased with further increasing Au/PDA/Fe_3_O_4_@C@PGC thickness, which attenuated the electron transfer. Therefore, 1.0 mg/mL was selected as the optimum concentration for the following experiments.

The concentration of K_3_[Fe(CN)_6_] has a marked impact on the signal output of the immunosensor. Therefore, the different concentrations of K_3_[Fe(CN)_6_] from 1 to 10 mM were used as basal solution. Figure 5(B) showed that the amperometric response increased with the increasing K_3_[Fe(CN)_6_] concentration and tended to decrease after 8 mM. Thus, 8 mM was selected as the optimal concentration.

### Assay performance

**Figure 6:**
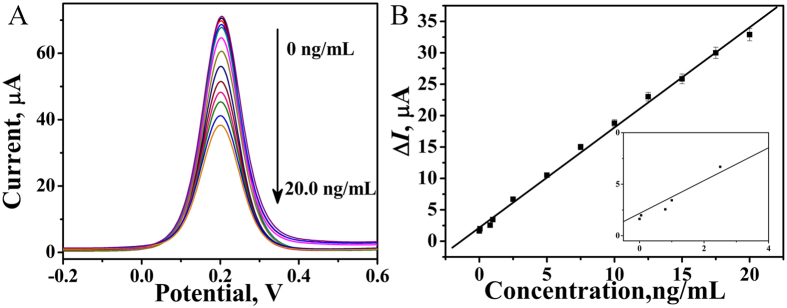
(**A**) Effect of CEA concentration on the DPV response of the immunosensor, (**B**) Calibration curve of the immunosensor toward different concentrations of CEA.

Differential pulse voltammetry (DPV) was used to evaluate the performance of the fabricated immunosensor under optimized conditions in Fig. 6(A). As shown in Fig. 6(B), the calibration plots between the peak current and the concentration displayed good linear relationships in the range of 0.001 ng/mL to 20.0 ng/mL for CEA with a regression equation of Δ*I* = 1.23 + 1.75 *c*, R = 0.988. The detection limit was estimated to be 0.33 pg/mL. The obtained detection limit was lower than that in some previous reports, such as 3 pg/mL^19^, 1 ng/mL^20^, 1.7 pg/mL^21^ and 1.0 ± 0.04 pg/mL^22^. The results showed enough sensitivity for detection of CEA.

In addition, the properties of immunosensor based on Au/PDA/Fe_3_O_4_@C@PGC nanocomposites for CEA detection were compared other electrochemistry immunoassay in Table 1. The results showed the assay performance of immunosensor was accepted.

### Selectivity and reproducibility of the fabricated immunosensor

In order to study the selectivity of the immunosensor, the squamous cell carcinoma antigen (SCCA), alpha fetal protein (AFP), vitamin C (Vc) and glucose found in the serum were selected as the interfering substances. In this paper, the 1 ng/mL CEA containing 20 ng/mL interfering substances was measured by the immunosensor. As indicated from Figure S1, when CEA coexisted with these sample interfering agents, no apparent signal change took place in comparison with that of only CEA. Meanwhile, significantly higher current response change was observed with the target CEA than with other biomarkers, which indicated that the proposed immunosensor revealed sufficiently selectivity for the detection of CEA. The relative standard deviation (RSD) of the measurements was 1.02%, which indicated that the selectivity of the immunosensor was acceptable.

In addition, the reproducibility also was an important parameter of the performance of the immunosensor. Therefore, five of identical batches of immunosensors were fabricated and measured for 1 ng/mL CEA in Figure S2. The RSD of the measurements was less than 3%, illustrating that the fabricated immunosensor has acceptable fabrication reproducibility.

### Analysis of real sample

In order to validate the feasible of the immunosensor, the recovery test with different concentrations of CEA in serum sample was carried out by standard addition method. The relative errors of the results were less than 5% for CEA detection in Table S1, and the recoveries were in the range of 98.4%–102.2%, suggesting good accuracy of the proposed method for real samples.

## Conclusions

A novel label-free immunosensor based on Au/PDA/Fe_3_O_4_@C@PGC nanocomposites was fabricated for the high sensitivity detection of CEA. Moreover, the fabricated immunosensor shows a wide linear range, a low detection limit, good specificity and acceptable reproducibility for the detection of CEA. In addition, the method is also applied to determine the CEA in the serum and shows the great application prospect in diagnosis and treatment of cancer.

## Additional Information

**How to cite this article**: Ji, L. *et al.* Preparation of Au-polydopamine functionalized carbon encapsulated Fe_3_O_4_ magnetic nanocomposites and their application for ultrasensitive detection of carcino-embryonic antigen. *Sci. Rep.* **6**, 21017; doi: 10.1038/srep21017 (2016).

## Supplementary Material

Supplementary Information
