# Supplementary material for: Preparation of Au-polydopamine functionalized carbon encapsulated Fe3O4 magnetic nanocomposites and their application for ultrasensitive detection of carcino-embryonic antigen
Source: Sci Rep. 2016 Feb 12;6:21017. doi: 10.1038/srep21017 (PMC4751493; doi:10.1038/srep21017)
Supplement: Supplementary Information [file srep21017-s1.pdf]

Supporting Information

Preparation of Au-polydopamine functionalized carbon encapsulated Fe_3O_4 magnetic nanocomposites and their application for ultrasensitive detection of carcino-embryonic antigen

Lei Ji[a], Tao Yan[a], Yan Li[a], Jian Gao[a], Qi Wang[b], Lihua Hu[a], Dan Wu[a], Qin Wei[a], Bin Du[a,*]

[a] Key Laboratory of Chemical Sensing & Analysis in Universities of Shandong, School of Chemistry and Chemical Engineering, University of Jinan, Jinan 250022, China

[b] School of Material Science and Engineering, University of Jinan, Jinan 250022, P.R. China

Lei Ji (E-mail: jilei_075098@163.com)

Tao Yan (E-mail: yantujn@163.com)

Yan Li (E-mail: sdjndliyan@163.com)

Jian Gao (E-mail: Gaojian8908@163.com)

Qi Wang (mse_wangq@ujn.edu.cn)

Lihua Hu (E-mail: hulihua1206@163.com)

Dan Wu (E-mail:wudan791108@163.com)

Qin Wei (E-mail: sdjndxwq@163.com)

Bin Du* (E-mail: dubin61@gmail.com)

*Corresponding author. Tel. + 86-531-82767370; fax: + 86-531-82765969.

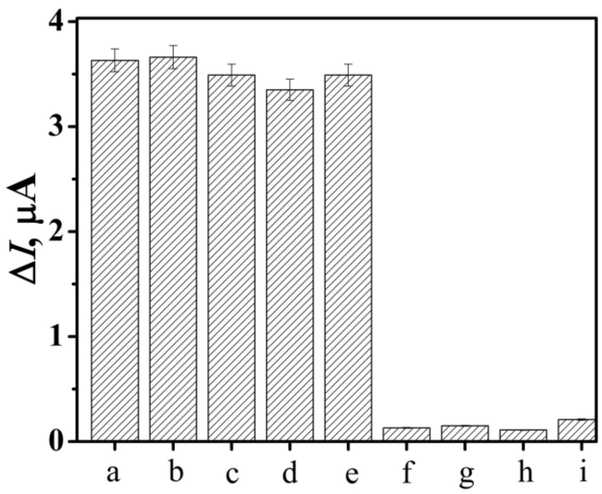

Figure S1 The study of the immunosensor's selectivity. (a) 1 ng/mL CEA； (b) 20 ng/mL of SCCA + 1 ng/mL CEA; (c) 20 ng/mL of AFP + 1 ng/mL CEA; (d) 20 ng/mL of Vc + 1 ng/mL CEA; (e) 20 ng/mL of glucose + 1 ng/mL CEA; (e) 20 ng/mL of SCCA; (e) 20 ng/mL of glucose; (e) 20 ng/mL of glucose; (e) 20 ng/mL of glucose; (inset: $\Delta I = I_0 - I_n$, in which I_n is the current response of the sensor to a series of different concentrations of CEA sample, I_0 is the the current response of the sensor to the blank sample containing no CEA) Error bar = RSD (n=5).

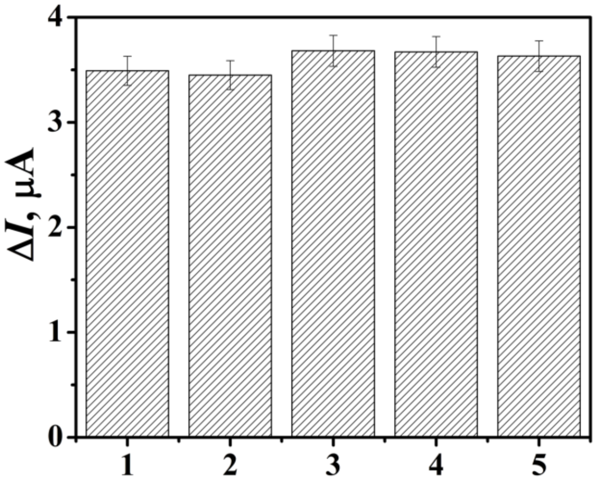

Figure S2 The study of the immunosensor's reproduction. Error bar = RSD (n=5).

Table S1. The results of the CEA determination in serum sample

Content of CEA in the serum (ng/mL^{-1})	The addition content (ng/mL^{-1})	The detection content (ng/mL)	RSD(%, n=5)	Recovery (%)
0.136	1.0	1.10,1.09,1.10,1.18, 1.20	4.5	99.8
	5.0	5.17,5.21,5.31,5.40, 5.15	2.0	102.2
	10.0	10.1, 9.54, 9.94, 10.0, 10.3	2.8	98.4
